# Case series of pediatric gastric trichobezoars: diagnostic challenges, management outcomes, and the imperative for psychiatric follow-up

**DOI:** 10.1097/RC9.0000000000000216

**Published:** 2026-02-16

**Authors:** Naser El-Mefleh, Ahmad Alsweed, Shaker Alhamedo, Ali Dani, Linah Kaf Alghazal, Imad Eddin Alshikh Saleh

**Affiliations:** a Pediatric Surgery Unit, Department of Surgery, Aleppo University Hospital, Aleppo, Syria; b Faculty of Medicine, University of Aleppo, Aleppo, Syria; c Department of Pediatric Surgery, American Hospital Dubai, UAE; d Department of General Surgery, Bab Al Hawa Hospital, Edleb, Syria; e Department of Gastroenterology, Al Hedaya Hospital, Edleb, Syria; f Department of Pediatric Surgery, Damascus University Children’s Hospital, Syria

**Keywords:** bezoars, gastric, pediatric, Rapunzel syndrome, trichophagia, trichotillomania

## Abstract

**Introduction and importance:**

Trichobezoar (TBZ) is a rare gastric mass composed of ingested hair, strongly linked to psychiatric conditions like trichotillomania (TTM) and trichophagia (TPH) that predominantly affect young females. Diagnosis, based on clinical suspicion, is best confirmed by CT or gastroscopy, though point-of-care ultrasound (POCUS) is a valuable initial tool. Management is ranging from endoscopic retrieval for small bezoars to surgical resection for large or complex cases. We present this series to highlight the diagnostic challenges, underscore the essential psychiatric dimension, and emphasize a recurrence risk of some cases.

**Case presentation:**

This report details five pediatric TBZ cases that all required surgical intervention for large masses (>10 cm). Presenting symptoms varied, encompassing anemia, abdominal discomfort, and failure to thrive. Diagnosis was achieved via POCUS, gastroscopy, CT, or MRI with subsequent surgical extraction performed via laparotomy or laparoscopy. Despite successful surgical outcomes, inconsistent psychiatric follow-up led to one case of recurrence following the resumption of hair-eating behavior.

**Clinical discussion:**

These cases underscore TBZ as a dual surgical and psychiatric condition. Successful management hinges on early imaging and a multidisciplinary approach. Clinicians should maintain a high index of suspicion for TBZ in children presenting with unexplained anemia and abdominal pain. Furthermore, psychiatric referral is imperative, even when hair-eating behaviors have ostensibly ceased, to address the underlying pathology and mitigate the significant risk of recurrence.

**Conclusion:**

The effective management of TB necessitates close collaboration between pediatric surgeons and psychiatrists from the point of diagnosis to ensure behavioral interventions are integrated with surgical care.

## Introduction

TBZ are accumulations of ingested hair that most commonly form in the stomach. Although they represent only 6% of all bezoars^[1]^, their strong association with psychiatric conditions, specifically TTM and TPH which are classified as obsessive-compulsive related disorders in the DSM-5, makes them clinically significant^[2]^. These behaviors predominantly affect young females, with a typical onset between 9 and 13 years of age and a population prevalence of 0.5–2.0%^[2]^. Some cases of TBZ may extend into the jejunum, ileum, and colon as a tail, which is referred to as Rapunzel syndrome^[3]^. Clinical presentation is often nonspecific, including a palpable epigastric mass (90%), abdominal pain (80%), nausea/vomiting (50%), or iron-deficiency anemia (IDA) from chronic mucosal irritation^[3,4]^. Left untreated, serious complications like gastric obstruction, perforation, or pancreatitis can arise^[5,6]^. Diagnosis combines clinical suspicion with imaging; contrast-enhanced CT is the diagnostic gold standard for defining the extent of the mass and identifying complications^[7,8]^, while POCUS can serve as an initial screening tool by revealing a characteristic hyperechoic mass with posterior shadowing^[9]^. Management is tailored to the size and complexity of TBZ, ranging from endoscopic techniques (e.g., fragmentation, Coca-Cola® dissolution) for small TBZ to laparotomy for large or complicated cases^[10,11]^. Given the strong psychiatric comorbidity, recurrence rates are high without addressing underlying disorders^[8]^. This report details a pediatric case series managed surgically and emphasizes the critical need for a multidisciplinary approach from diagnosis onwards. The paper agreed with the PROCESS checklist^[12]^.

**HIGHLIGHTS**

- Surgery is often necessary for large pediatric trichobezoar (TBZ) via laparotomy and laparoscopy.
- A high index of suspicion is key to diagnosis of TBZ, as presenting symptoms are often non-specific.
- The series demonstrates the diagnostic tools including POCUS, gastroscopy, CT, or MRI for preoperative planning and to rule out complications.
- Multidisciplinary care model is imperative from the moment of diagnosis to prevent recurrence.

## Methods and results of case series

This retrospective, multi-institutional case series (*n* = 5). Pediatric patients diagnosed with and surgically treated for TBZ across four institutions. For each case, we collected data on patient demographics, clinical presentation, diagnostic workup, surgical management, complications, and psychiatric follow-up. All patients received standard general anesthesia and preoperative antibiotic prophylaxis (ceftriaxone and metronidazol). The primary aim was to analyze the variable clinical presentations, highlight diagnostic pathways, and evaluate the critical role of psychiatric comorbidity in recurrence.

### Case 1: The overlooked habit

**Figure 1.**
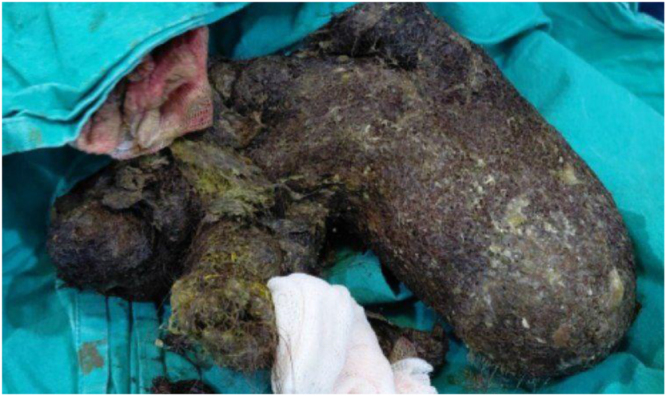
Gross specimen shows the typical characteristics of a mature TBZ.

A 9-year-old girl was referred to gastroenterologist for progressive IDA and anorexia, notably without classic obstructive symptoms such as vomiting, weight loss, or significant pain. Her history was significant for Trichotillomania (TTM) with onset at 2 years of age, which evolved into Trichophagia (TPH) following the birth of a younger sibling. Interventions such as head shaving had been attempted, and the family reported the hair-eating behavior had ceased spontaneously by 6 years of age. Physical examination was notable for pallor and a firm, non-tender epigastric mass. Laboratory studies confirmed severe microcytic anemia (hemoglobin 7.8 g/dl). Gastroscopy (Supplemental Digital Content Video 1, available at: http://links.lww.com/IJSCR/A8) identified a massive, dark TBZ occupying the gastric lumen extend through pylorus (Rapunzel syndrome). A subsequent CT scan confirmed the findings. Due to large size of TBZ (>10 cm), endoscopic removal was considered unsafe. The patient consequently underwent laparotomy with gastrotomy, and the TBZ was extracted (Fig. 1). Her recovery was uncomplicated, with enteral nutrition initiated on 5th day and discharge on 6th postoperative day. Psychiatric evaluation confirmed diagnoses of TTM and TPH, and cognitive-behavioral therapy (CBT) was recommended. The family attended three sessions but was subsequently lost to follow-up.

### Case 2: The incidental finding

**Figure 2.**
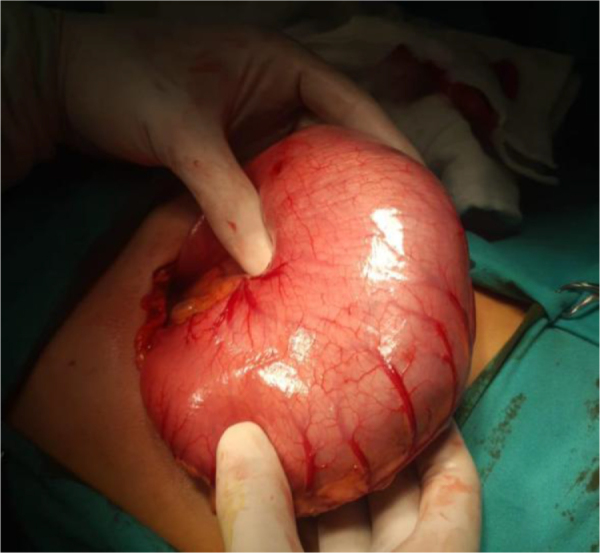
Intraoperative view illustrates the mechanical gastric distension caused by a large TBZ.

**Figure 3.**
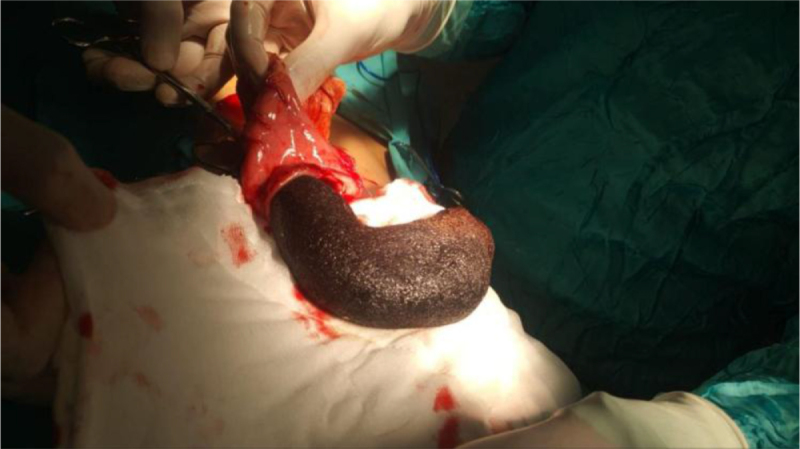
Intraoperative extraction of TBZ through a gastrotomy incision as *en bloc* removal.

A previously healthy 7-year-old girl presented with a history of chronic abdominal discomfort and early satiety. Clinical evaluation revealed failure to thrive and a documented history of TPH. Physical examination identified a firm, non-tender epigastric mass, and laboratory workup showed only mild microcytic anemia (hemoglobin 10.2 g/dl, MCV 74 fL). POCUS was diagnostic, revealing a large hyperechoic intragastric mass with posterior acoustic shadowing consistent with a gastric TBZ. She underwent a supraumbilical laparotomy because of large size. Intraoperative findings appeared TBZ occupied approximately 80% of the gastric lumen (Fig. 2), and it was removed *en bloc* via gastrotomy (Fig. 3). Her recovery was uneventful, with enteral nutrition initiated on 3rd day and discharge on 5th postoperative day. The family was strongly advised to pursue psychological consultation however they declined, stating the habit had ceased. They agreed to seek help only if symptoms of the behavior repeated. At 21-month follow-up, there was no evidence of recurrence.

### Case 3: The recurrence

**Figure 4.**
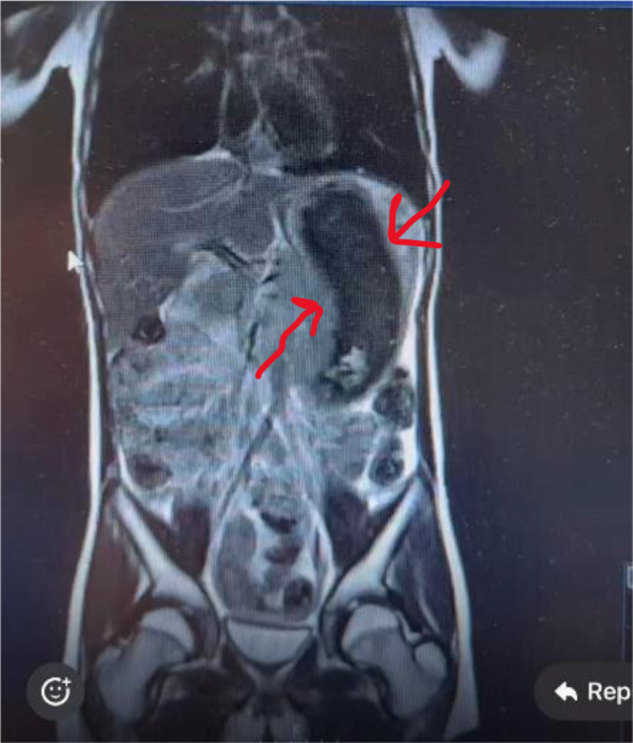
Coronal MRI view shows large TBZ as a well-defined, intragastric mass extending vertically, occupying a significant portion of the gastric lumen. The mass has a heterogeneous signal, suggestive of hair content.

**Figure 5.**
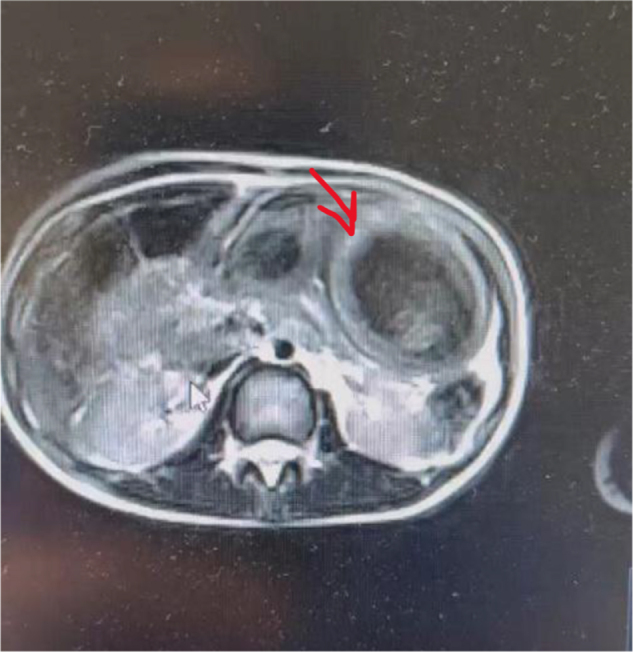
Axial MRI view confirming the intragastric location and internal heterogeneity of TBZ. The mass appears mottled and low signal intensity.

**Figure 6.**
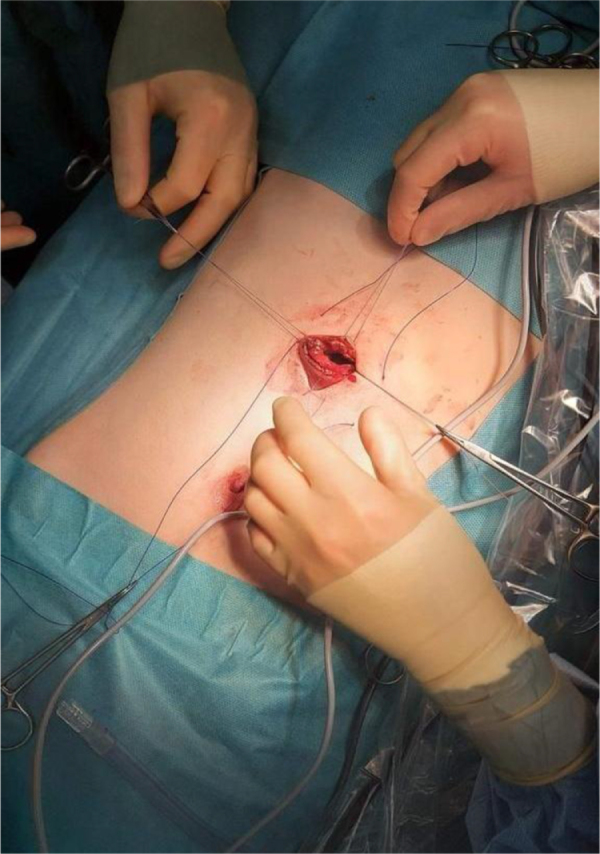
Intraoperative view shows the minimally invasive surgical approach for extraction.

**Figure 7.**
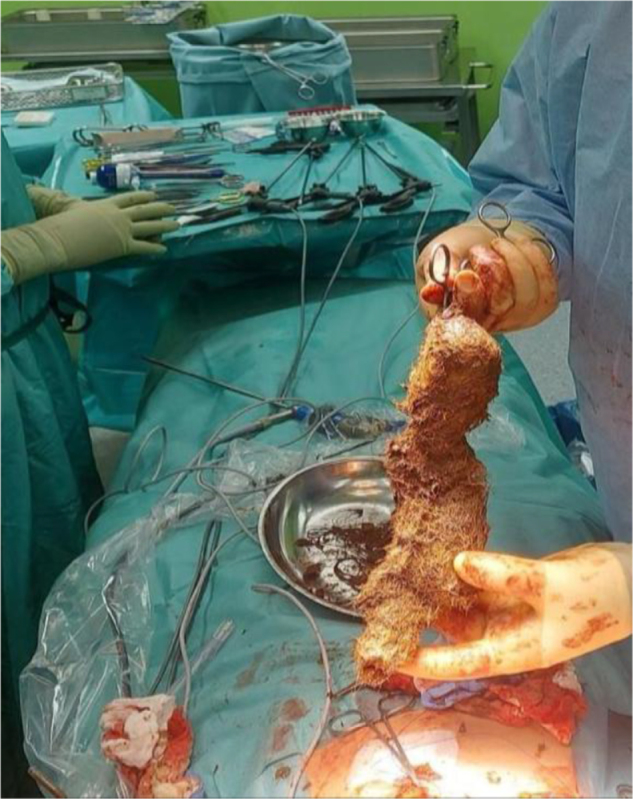
Intraoperative image depicts the successful extraction of a large bezoar via a minimally invasive technique.

An 8-year-old girl presented with abdominal pain, distension, and palpable masses. Initial laboratory studies were unremarkable. Given concern for lymphoma, an urgent MRI was obtained (Figs 4 and 5), which unexpectedly identified a large gastric TBZ. MRI was selected over CT due to its superior soft-tissue characterization and to avoid ionizing radiation in a child, according to the institute policy. Re-evaluation recognized a previously long-standing history of TPH, and the patient also exhibited fine scalp hair and avoidant traits. Endoscopy revealed that the stomach was nearly filled with the TBZ. She underwent a laparoscopy with exteriorization of the stomach through a small epigastric incision (Fig. 6) and intact extraction of the TBZ (Fig. 7). Postoperative psychiatric follow-up was arranged, but compliance was poor, with the family attending only one session. Fourteen months later, she re-presented with a recurrent abdominal mass and resumed TPH, confirming a new TBZ that required a second laparoscopic removal. After this procedure, CBT was tried for 6 months before losing the follow-up.

### Case 4: The Straightforward Course

A 5-year-old girl was evaluated for nonspecific abdominal pain. While physical examination and initial laboratory findings were unremarkable, POCUS identified a gastric mass, leading to a gastroenterology referral. Endoscopy confirmed the presence of a gastric TB. It was not suitable for endoscopic removal or dissolution therapy, and the patient successfully underwent a laparoscopic extraction using the technique previously described in Case 3. Her recovery was well, with oral intake tolerated on the second postoperative day. A concurrent psychiatric referral diagnosed TPH. The patient and family participated in weekly play therapy and parental guidance sessions for 1 year, demonstrating full adherence to the treatment plan. Through 1 year of combined surgical and psychiatric follow-up, she demonstrated excellent progress and showed no evidence of recurrence.

### Case 5: The complex presentation

**Figure 8.**
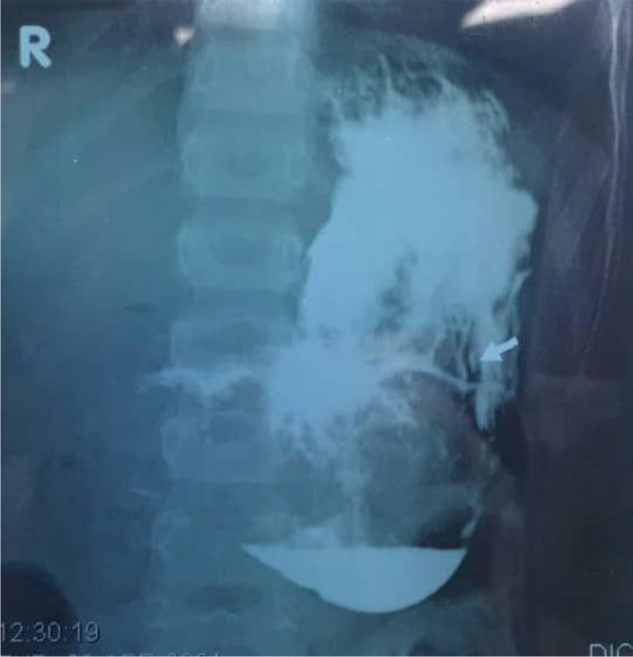
Upper GI series demonstrating gastric outlet obstruction caused by TBZ. Key features include a large filling defect occupying the gastric lumen and delayed passage of contrast into the duodenum.

**Figure 9.**
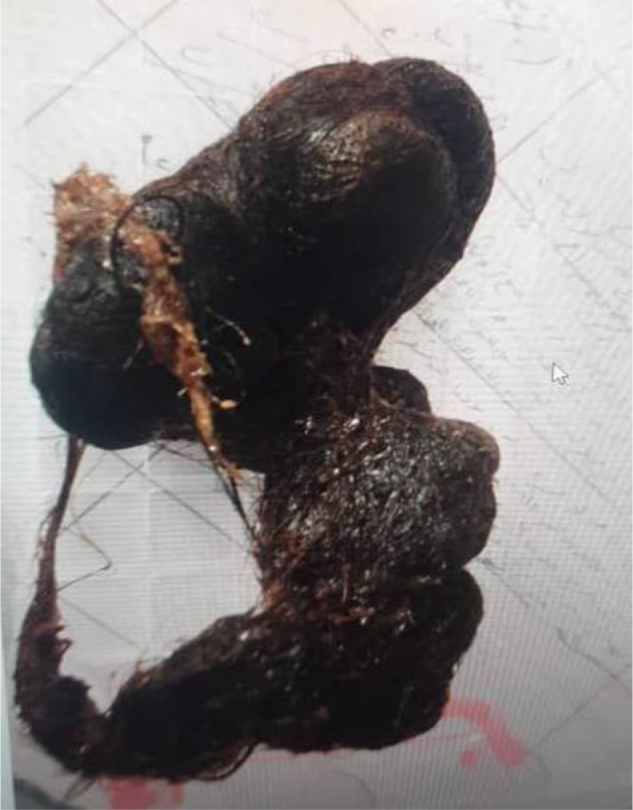
Visual documentation of a very large TBZ composed of a solid mass of hair, weighed approximately 500 grams.

A 7-year-old girl with a history of TPH since 2 years of age and a prior appendectomy was admitted with vomiting suggestive of gastric outlet obstruction. Notably, she was not anemic. POCUS indicated an echogenic gastric mass, and an upper GI series (Fig. 8) showed significant filling defects, delayed contrast passage, and inflammatory mucosal changes. Gastroscopy confirmed a firm TB extending from cardia to pylorus, accompanied by multiple gastric ulcers. Laparotomy revealed a large TBZ weighing approximately 500 grams (Fig. 9), which was extracted via gastrotomy. Due to the ulcerations, the gastrotomy was reinforced with an omental (Graham) patch. Her 7-day hospitalization involved a gradual, well-tolerated advancement of enteral nutrition. Psychiatry was consulted during admission, confirming the TPH diagnosis. She was discharged with CBT management of her underlying TPH.

## Discussion

The term “bezoar” was first used by Richard Quain in 1854 to describe a mass found in the stomach^[13]^. Bezoars are intraluminal concretions formed by the accumulation of indigestible materials, such as hair, fruit fibers, or dairy products, within the gastrointestinal tract. A specific type, the TBZ, develops when ingested hair accumulates in the gastric folds and fails to digest^[14]^. TBZ often present a diagnostic challenge and patients may remain asymptomatic or only mild abdominal pain leading to delayed medical attention. The clinical presentation depends on the size and location of the mass^[14]^. Consequently, hair-eating behavior often continues until the development of severe pain, obstructive symptoms or serious complications^[15–17]^. Although TTM and TPH are common in these patients, only about 0.5% of individuals with these behaviors develop a gastric TBZ^[18,19]^. A key unanswered question remains the precise quantity of hair or duration of ingestion required to progress to a symptomatic TBZ. Early-onset TTM, defined by symptom onset between ages 2 and 10 years, often resolves spontaneously^[20]^. However, as illustrated by our cases (particularly cases 1 and 3), a reported history of resolved TPH should be interpreted with caution and does not preclude the presence of a large TBZ. Due to their young age, children with this condition are typically unable to conceal the behavior, leading to early recognition by families and prompt intervention. Treatment compliance is generally higher in this group, as comorbid psychiatric conditions are less common at this age^[20]^.

A review of the literature reveals no standardized radiological approach for diagnosis. Diagnostic imaging includes CT (the gold standard for diagnosing), endoscopy (both a diagnostic and therapeutic tool), and POCUS (frequently the first-line imaging modality)^[8,21,22]^. In our series, POCUS served as an effective tool in (cases 2, 4, and 5), while CT in (Case 1). MRI (Case 3) were used for detailed preoperative planning and to avoid radiation according to institute protocol.

There is no consensus on the optimal method for TBZ removal. However, the effective management involves a combination of medical, endoscopic, and surgical approaches depending on the size, location, and severity of complications^[8]^. As our cases illustrate, addressing underlying psychological disorders (TTM and TPH) is essential to prevent recurrence. Endoscopic management remains technically challenging for TBZ^[23]^. However, advancements in endoscopic techniques have led to improved outcomes, with success rates increasing even giant TB^[8,24]^. Smaller TB may be managed endoscopically with techniques like Coca-Cola dissolution or argon plasma coagulation^[10,11,25]^.

Surgical treatment is the most effective approach. While laparotomy is still the gold standard for large or complicated TBZ, laparoscopy offers significant advantages as minimal invasive technique with benefits of reduced morbidity and a faster recovery^[8]^. Intraoperative gastroscopy can be a valuable adjunct to ensure complete removal, particularly for assessing residual fragments or a distal tail in Rapunzel syndrome^[26]^. In our series (Table 1), the surgical approach (open vs laparoscopic-assisted) was determined by TBZ size, and surgeon experience. No postoperative wound complications occurred in our series aligns with the literature, despite the contaminated nature of the surgery^[27]^.

**Table 1:** Clinical characteristics and outcomes of pediatric trichobezoar cases

Case	Age/Sex	Key symptoms	Imaging findings	Size (cm)	Surgical approach	Complications	Psychiatric follow-up
1	9 F	IDA (Hb 7.8 g/dl), epigastric mass	CT: Large gastric TBZ	12 × 8	Laparotomy	None	Lost to follow-up after 3 sessions (~3 months)
2	7 F	Failure to thrive, mild anemia (Hb 10.2 g/dl)	US: Hyperechoic gastric mass	11 × 6	Laparotomy	None	Declined by family
3	8 F	Abdominal pain, distension	MRI: large gastric TBZ	8 × 5	Laparoscopy	Recurrence	2 sessions only, recurrence at 14 months
4	5 F	Nonspecific abdominal pain	US + Endoscopy: Gastric TBZ	7 × 4	Laparoscopy	None	Complete adherence for 12 months
5	7 F	Gastric outlet obstruction	CT: TBZ with duodenal extension	15 × 10	Laparotomy	Gastric ulcers	1 initial visit, then lost to follow-up

Each surgical technique used in the treatment of TBZ has its own advantages and limitations (Table 2)^[8]^.

**Table 2:** Comparison of surgical methods for trichobezoar

Surgical method	Advantages	Disadvantages	Indications
Laparotomy	Ensures complete TBZ extraction Lower recurrence risk	Higher morbidity Longer recovery time Increased risk of wound infection	Large TBZ (>10 cm) Presence of complications
Laparoscopy	Minimally invasive Reduced postoperative pain Lower risk of wound infection Better cosmetic outcome	Technically challenging Prolonged operative time Risk of peritoneal contamination	Moderate-sized TBZ (5–10 cm) No significant complications
Hybrid approach (laparoscopy + endoscopy)	Combines advantages of both techniques Improved visualization Allows controlled fragmentation and extraction Minimizes peritoneal contamination risk	Requires advanced expertise Prolonged surgical time Risk of incomplete removal if not well-coordinated	Cases where endoscopic assistance can facilitate extraction TBZ that require fragmentation but can be managed with minimal invasive surgery

Psychiatric intervention is essential in the management to prevent recurrence. CBT particularly habit reversal training (HRT), remains the most evidence-based first-line intervention^[8,28]^. Relapse remains common, especially when follow-up is unstructured or prematurely discontinued. Recurrence rates range 30–67% in the absence of ongoing psychiatric support^[2,8]^. N-acetylcysteine has shown promise in adults with TTM and has good tolerability in pediatric patients, though its efficacy may be limited in those with automatic hair-pulling behaviors^[29,30]^.

Strategies to mitigate recurrence include: a multidisciplinary model from diagnosis, parental education, a minimum psychiatric follow-up of 12 months postoperatively^[8]^, and school-based screening for TTM in high-risk populations^[15]^. In our series, the critical importance of consistent psychiatric follow-up is clearly demonstrated. The only documented case of recurrence (case 3) followed poor engagement with recommended psychiatric care. Conversely, case 1 and 2, where the family declined formal consultation, highlights a critical gap; despite surgical success, the underlying behavioral risk remains unaddressed, creating potential for future recurrence.

## Limitations

Its generalizability is constrained by the small sample size and its retrospective, descriptive nature. The cases were gathered from different centers over a non-specified timeframe, leading to variability in imaging protocols, surgical techniques, and follow-up duration, which precluded statistical analysis of risk factors for recurrence or complications. Future prospective studies with standardized, multidisciplinary protocols are needed.

## Conclusion

Effective management of pediatric TBZ requires early imaging and a multidisciplinary approach integrating surgical and psychiatric care. While minimally invasive techniques offer advantages in selected cases, surgery alone is insufficient without long-term psychological support. Emphasis on early psychiatric referral is imperative, even when hair-eating behaviors have ostensibly ceased, to address the underlying pathology, prevent recurrence, and improve long-term outcomes.

## Data Availability

Not applicable.
